# Identification of appropriate reference genes for human mesenchymal stem cell analysis by quantitative real-time PCR

**DOI:** 10.1007/s10529-014-1652-9

**Published:** 2014-09-02

**Authors:** Xiuying Li, Qiwei Yang, Jinping Bai, Yali Xuan, Yimin Wang

**Affiliations:** 1 The Central Laboratory, China-Japan Union Hospital, Jilin University, 126 Xiantai Street, Changchun, 130033 Jilin China; 2 Department of Pathology, The Key Laboratory of Pathobiology, Jilin University, Ministry of Education, 126 Xinmin Street, Changchun, 130021 Jilin China; 3 Princess Margaret Hospital, 610 University Ave, Toronto, ON M5G 2M9 Canada; 4 Jilin Zhongke Bio-engineering Co., Ltd, Building 2, 3355 Guigu Street, Changchun, 130012 Jilin China

**Keywords:** Gene expression, Mesenchymal stem cells, Reference genes, Reverse transcription-PCR

## Abstract

Normalization to a reference gene is the method of choice for quantitative reverse transcription-PCR (RT-qPCR) analysis. The stability of reference genes is critical for accurate experimental results and conclusions. We have evaluated the expression stability of eight commonly used reference genes found in four different human mesenchymal stem cells (MSC). Using geNorm, NormFinder and BestKeeper algorithms, we show that beta-2-microglobulin and peptidyl-prolylisomerase A were the optimal reference genes for normalizing RT-qPCR data obtained from MSC, whereas the TATA box binding protein was not suitable due to its extensive variability in expression. Our findings emphasize the significance of validating reference genes for qPCR analyses. We offer a short list of reference genes to use for normalization and recommend some commercially-available software programs as a rapid approach to validate reference genes. We also demonstrate that the two reference genes, β-actin and glyceraldehyde-3-phosphate dehydrogenase, are frequently used are not always successful in many cases.

**Electronic supplementary material:**

The online version of this article (doi:10.1007/s10529-014-1652-9) contains supplementary material, which is available to authorized users.

## Introduction

Mesenchymal stem cells (MSC) are characterized by their ability to differentiate into several mesenchymal and non-mesenchymal lineages (Keating 2006) and are potential candidates for tissue regeneration and repair. MSC are self-renewing cells derived from different sources including bone marrow and other adult and fetal tissues. Among these tissues, adipose tissue (Kern et al. 2006), Wharton’s jelly of the umbilical cord (Anzalone et al. 2010) and placenta tissue (Manochantr et al. 2013) have been regarded as prospective alternatives to bone marrow. The most important point is the comparison of commercially-available MSC with more primitive populations of MSC, such as bone marrow MSC, adipose tissue MSC, Wharton’s jelly MSC and placenta tissue MSC.

Among the commonly-used techniques, quantitative reverse transcription-PCR (RT-qPCR) is currently the gold standard for the quantification of steady-state mRNA levels due to its accuracy and sensitivity (Ginzinger 2002). However, there is increasing evidence suggesting that expression of these types of genes vary between cell types and experimental conditions. Therefore it is essential to identify the reference gene prior to its use for normalization during specific experimental set ups. But there is no single reference gene that is universal and completely constant. In contrast, there is increasing evidence emerged that expression of common used reference genes varies greatly among different cell types and experimental conditions (Schmittgen and Zakrajsek 2000). Thus, the selection of a suitable reference gene for specific study is a prerequisite for RT-qPCR assay to obtain reliable result. The aim of this study was to identify and assess the stability and reliabilities of eight commonly used reference genes in MSC in RT-qPCR. Eight common reference genes were selected and used for this study and the expression stability was analyzed by geNorm, NormFinder and BestKeeper respectively.

## Materials and methods

### Isolation and expansion of mesenchymal stem cells (MSC)

MSC were isolated from four human tissue sources: adipose tissue, bone marrow, Wharton’s jelly from umbilical cord and placenta. Wharton’s jelly from umbilical cord and placenta were from normal caesarean birth and bone marrow and adipose tissue samples were obtained from healthy volunteer donors. All individuals gave written informed consent and the study was approved by the Ethical Committee of China-Japan Union Hospital, Jilin University. Hyalurodinase and collagenase were used to digest the umbilical cord after the outside skin was removed. Placenta and adipose tissue were digested by collagenase only. Bone marrow MSC was obtained by bone marrow adherence culture. After processing, MSC was plated in a culture flask with α-MEM medium supplemented with 10 % (v/v) FBS and 1 % penicillin/streptomycin. The culture was maintained at 37 °C with saturated humidity and 5 % CO_2_. The sample number was five each for all four populations of MSCs.

### Quantitative reverse transcription-PCR

Total cellular RNA was extracted and RT-qPCR were performed as described previously by Li et al. (2014). The assay efficiency was measured by four points of serial dilution of cDNA (1:10; 1:100; 1:1,000 and 1:10,000) of pooled samples based on the slope of the standard dilution curve: Efficiency = [10^1/−slope^ − 1] × 100 %, where slope represents the slope of the linear regression (Ginzinger 2002). Mean cycle threshold (Ct) values of each tenfold dilution were schemed against the logarithm of the cDNA dilution factor. The sample number was five each for all four populations of MSCs.

All primers were synthesized by Sangon Biotech (Shanghai, China) with Tm at 60 °C. The full name and accession number of the reference genes, primer sequences and corresponding to amplicon sizes are listed in Supplementary Table 1.

### Data analysis

The data were analyzed using the software geNorm, NormFinder, and BestKeeper. The geNorm algorithm provides a measure of gene expression stability (M value) and creates stability ranking via a stepwise exclusion of the least stable. The lowest M value corresponds to the most stable reference gene (Vandesompele et al. 2002).

NormFinder calculates expression stability values via an analysis of variance (ANOVA)-based model. The lowest value that is obtained indicates the highest expression stabilities. NormFinder evaluates not only the overall expression variation of the reference genes, but also the variation between sample subgroups. The output consists of a stability value based on both intra- and inter-group expression variations in NormFinder.

BestKeeper estimates the expression variability of reference genes by calculating the raw Ct data and analyzes reference gene stability based on the standard deviation (SD) and coefficient of correlation (*r*) of all the reference genes. SD values are obtained from the Ct value of each reference gene, and *r* values are calculated via Pearson’s pair-wise correlation analyses between each reference gene and the geometric mean of the Ct values. None of the reference genes included in this study had SD values higher than 1.0, indicating that all these genes were suitable candidate reference genes.

## Results and discussion

### Analyses of RNA quality and primer efficiency

**Fig. 1:**
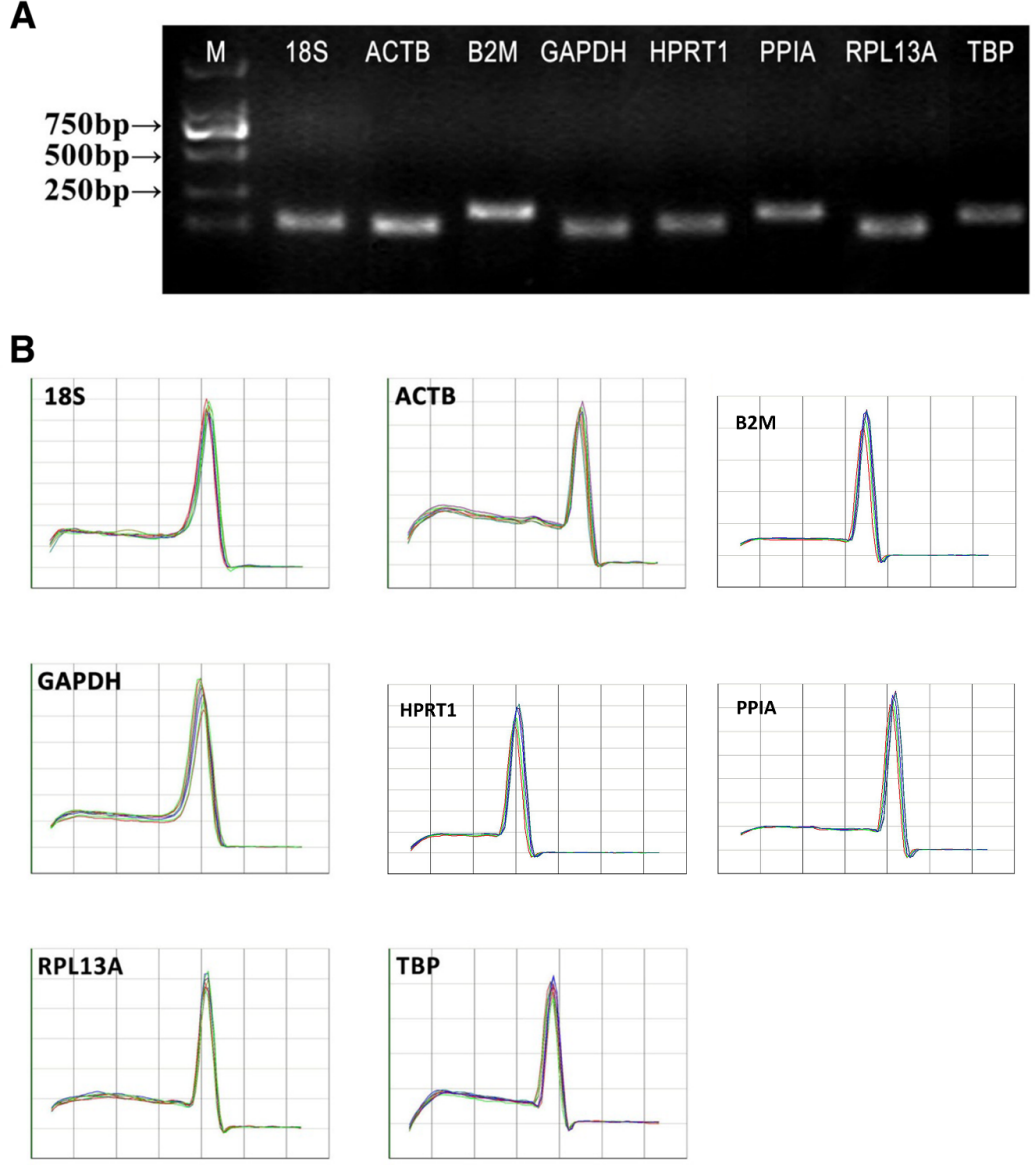
Specificity of primers and amplicon length. The PCR amplification products for the eight reference genes were analyzed by agarose gel electrophoresis and dissociation curves. **a** PCR products were run on a 2 % agarose gel. A single band at the anticipate product size indicates that the PCR product is specific. M: size markers. **b** Dissociation curves for the PCR products. The single peak represents a specific PCR product

The A_260/280_ ratio for the isolated RNA was 1.85–2. All the PCR assays produced single amplicons with the expected sizes, as shown by the absence of nonspecific bands in the electrophoresis gels and by the presence of a single sharp peak in the dissociation curve analysis (Fig. 1). The PCR efficiency of each primer pair was verified by standard dilution curve analysis using the cDNA samples in a tenfold serial dilution. All the primer pairs demonstrated efficiency values that ranged from 80.87 to 108.02 %, with correlation coefficients higher than 0.97.

### Expression levels of the reference genes

**Fig. 2:**
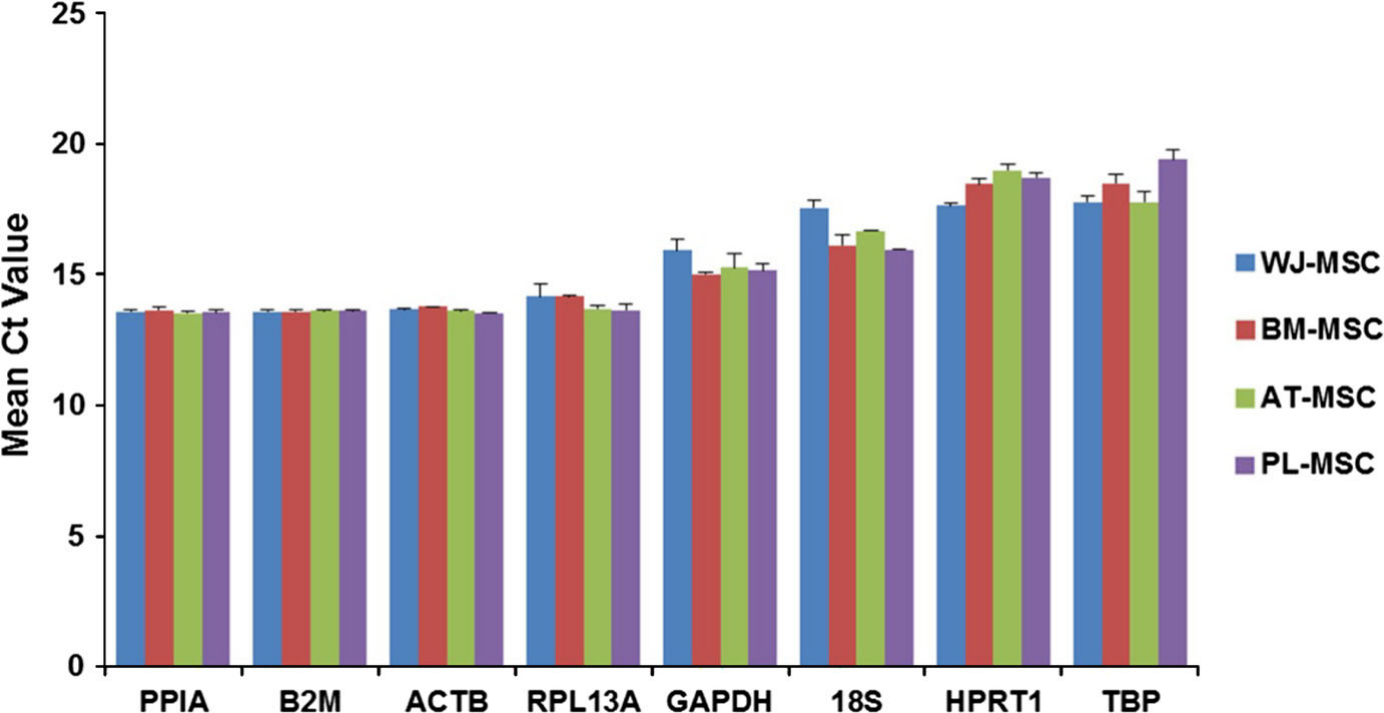
Expression levels of the reference genes among four cell groups. Mean Ct values are shown (mean ± SD; n = 5). BM-MSC represents bone marrow MSC; AT-MSC represents adipose tissue MSC; WJ-MSC represents Wharton’s jelly of the umbilical cord MSC; PL-MSC represents placenta tissue MSC

A suitable reference gene is one that is expressed at relatively high but stable levels (Huggett et al. 2005). The expression levels of the eight reference genes among the different tissues are shown in Fig. 2. The mean Ct values for the eight genes were between 13.6 ± 0.08 (peptidyl-prolylisomerase A, PPIA) and 19.4 ± 0.36 (TATA box binding protein, TBP). The variations in transcript expression levels were largest for 18S ribosomal RNA (18S) and TBP (1.62 and 1.63 cycles, respectively), and much lower for β-2-microglobulin (B2M) and PPIA (0.04 and 0.09 cycles, respectively). When expression alterations between different cell types are considered extra care should be taken to choose reference genes that are expressed at similar relative levels among these cell types.

### B2M and PPIA are the most stably expressed reference genes in human MSC

**Fig. 3:**
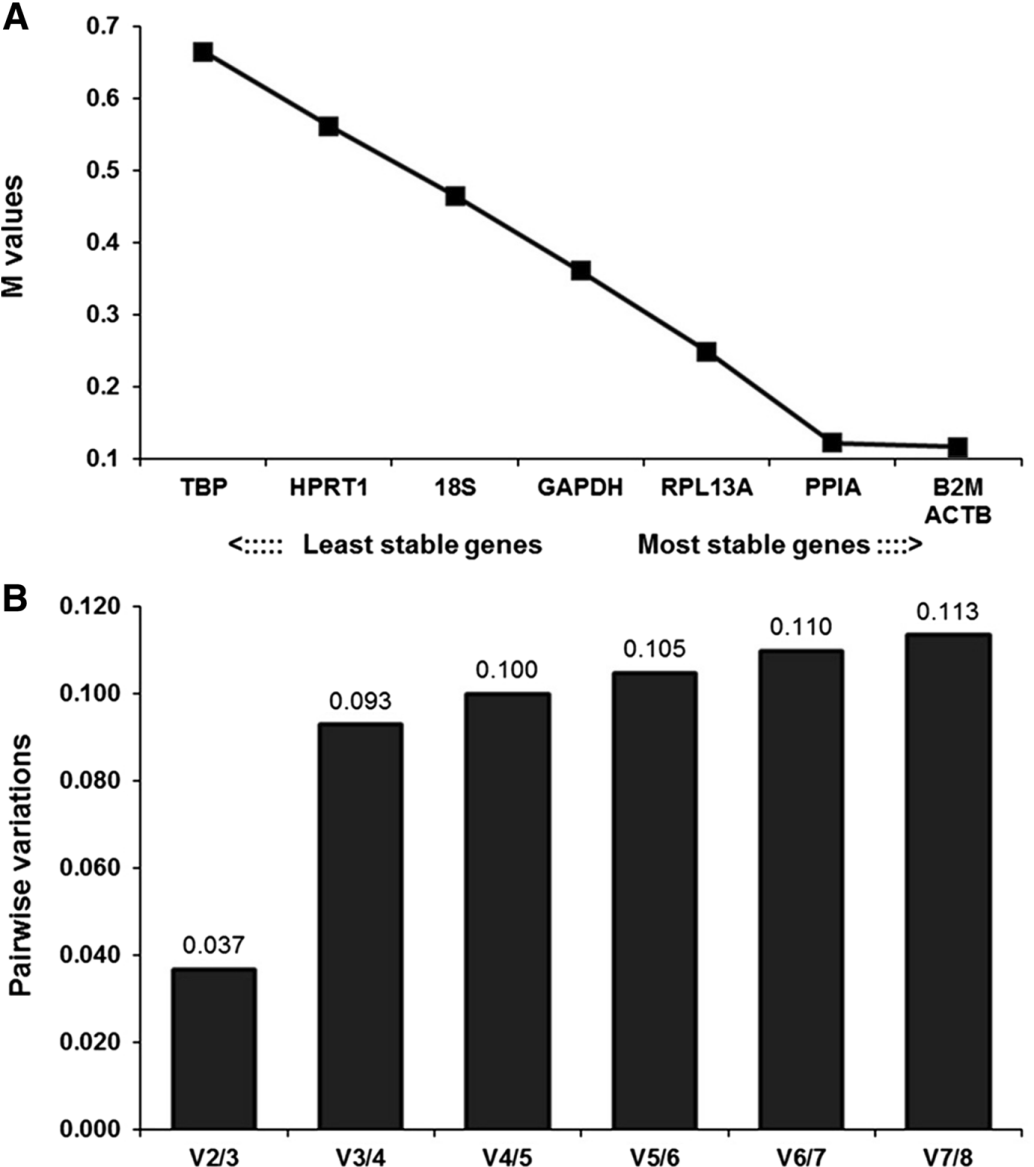
Ranking and determination of optimal number of reference genes using geNorm. **a** Expression stability measure (M) of the eight reference genes analyzed. Ranking of the genes according to their expression stability is indicated on the x-axis; low M values indicate high expression stability. **b** Optimal number of reference genes for normalization based on their V values. The V value defines the pair-wise variation between two sequential normalization factors calculated by geNorm

The geNorm, NormFinder, and BestKeeper results showed that the expression stability of the reference genes was slightly different. The differences in stability were probably caused by the different algorithms used in the three software packages (Chang et al. 2012). Based on geNorm (Fig. 3), each of the reference genes had an average expression stability value (M) less than 1.5, indicating that none of genes were unreliable. Among the eight genes, β-actin (ACTB) and B2M were the optimal reference genes, followed by PPIA, and TBP was ranked as the most unstable gene. Although geNorm recognized the most stable reference genes, B2M and ACTB, it still recommended the use of two or more reference genes for accurate normalization and to exclude potential instability (Hellemans et al. 2007). The pairwise variation coefficient (shown as V in Fig. 3b) was used to determine the optimal number of reference genes. If geNorm generates *n* as the optimal number of reference gene, when the value of V*n*/*n* + 1 dropped below 0.15, the genes with the lowest *n* values of M became the final choice. Because V2/3 was less than 0.15 (Fig. 3b), the optimal number of reference genes in this experimental situation was two (B2M and ACTB), because they had the lowest M values (Fig. 3a).

**Fig. 4:**
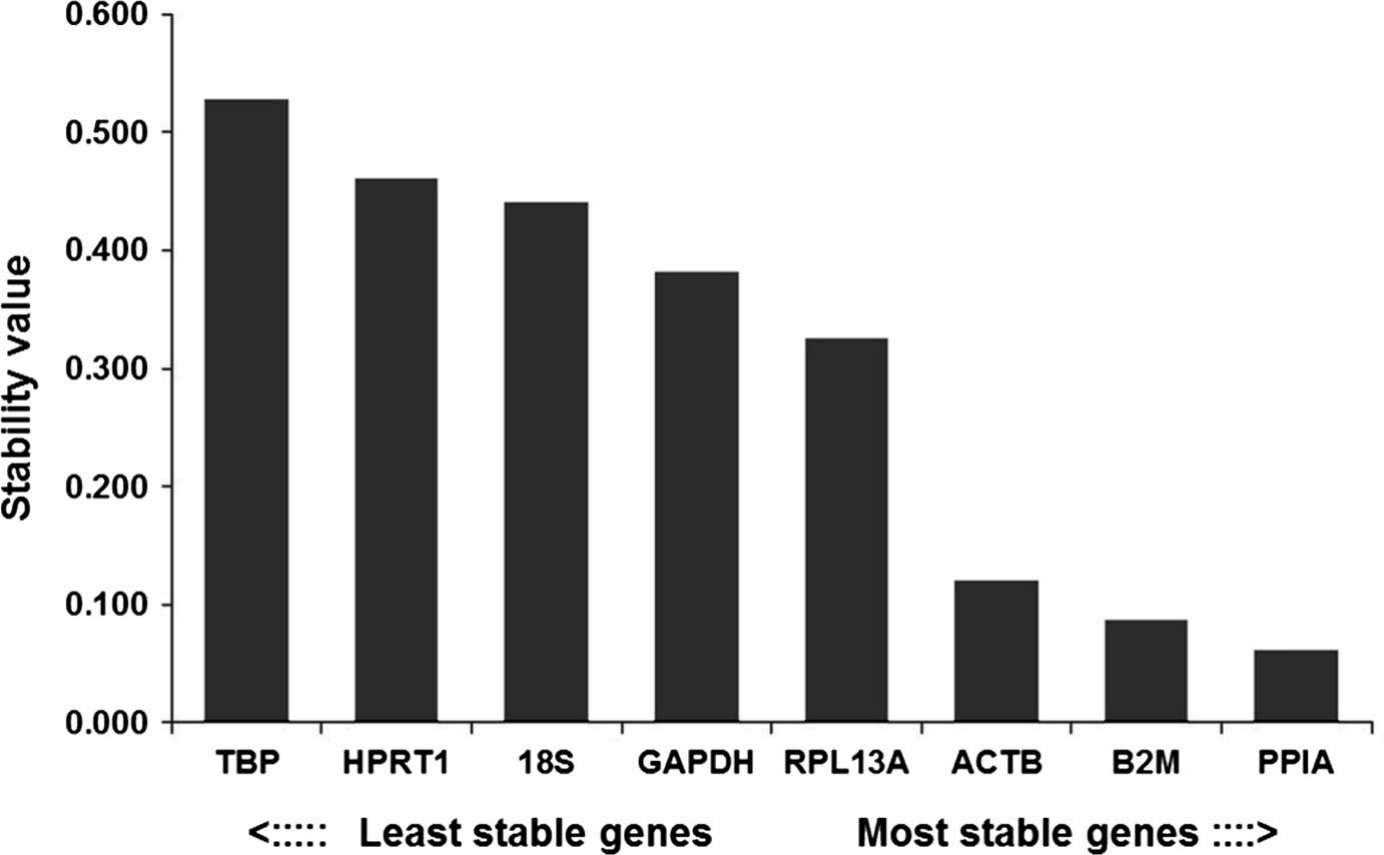
Expression stability values of the reference genes calculated using NormFinder. Ranking of the genes according to their expression stability is indicated on the* x*-axis; low stability values imply stable expression

Similarly, based on the NormFinder results (Fig. 4), PPIA was the best gene, followed by B2M and ACTB. When the intergroup variations were considered, the best combination was PPIA and B2M. The combined stability value (0.07) was only a little higher than the stability value for PPIA (0.062), indicating that PPIA could be used independently as a reference gene for relative quantitation.

**Fig. 5:**
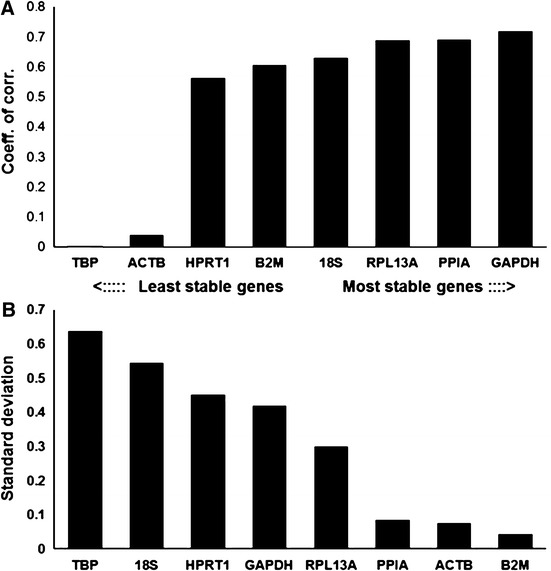
Evaluation of reference genes using BestKeeper. **a** Correlation coefficient (*r*) values of the reference genes evaluated by BestKeeper. Ranking of the genes according to their expression stability is indicated on the* x*-axis; low stability values imply stable expression. **b** Standard deviation (SD) values of the reference genes

The results of the BestKeeper analysis indicated that the lowest variations displayed the highest stability (Fig. 5). Variations of SD > 1 were considered unacceptable, implying that all eight genes were credible reference genes because the SDs were all below 0.7. Because high *r* values suggest stable expression, glyceraldehyde-3-phosphate dehydrogenase (GAPDH) was found to be the most stable reference gene while TBP was the most unstable reference gene.

In our study, GAPDH was not the most stable reference gene identified in the geNorm and NormFinder results. Although GAPDH is a widely used reference gene, the result shows that, of the eight normalization genes tested, GAPDH had the highest gene instability values among the four kinds of MSCs. We conclude that GAPDH is not a reliable reference genes for the normalization of RT-qPCR data in human MSC research, contradictory to its continued use in this field of research (Raicevic et al. 2011). In previous studies, ACTB has been commonly used as a reference gene to evaluate target gene expression levels (Najar et al. 2010). The geNorm analysis found that ACTB was the most stable gene among the eight reference genes; while ACTB was the second and third most stable reference gene in bestkeeper and NormFinder respectively (Table 1). Therefore, ACTB may not be the best reference gene for RT-qPCR of human MSC.

**Table 1:** Expression stability of 8 RGS in different groups evaluated by BestKeeper

	PPIA	RPL13A	GAPDH	HPRT1	18S	TBP	B2M	ACTB
Geo Mean (Ct)	13.57	13.9	15.33	18.44	16.56	18.35	13.59	13.63
Ar Mean (Ct)	13.57	13.9	15.34	18.45	16.57	18.36	13.59	13.63
Min (Ct)	13.48	13.39	14.76	17.54	15.69	17.37	13.48	13.49
Max (Ct)	13.74	14.63	16.33	19.22	17.86	19.76	13.67	13.77
Std dev (±Ct)	0.085	0.3	0.42	0.452	0.545	0.638	0.043	0.076
CV (% Ct)	0.622	2.15	2.734	2.452	3.287	3.477	0.316	0.557
Coeff of corr. (r)	0.69	0.688	0.719	−0.564	0.631	0.002	−0.606	−0.041

*geo Mean* (*Ct*) the geometric mean of Ct, *ar Mean* (*Ct*) the arithmetic mean of Ct, *Min* (*Ct*) *and Max* (*Ct*) the extreme values of Ct, *stddev* (±*Ct*) the standard deviation of the Ct, *CV* (*%Ct*) the coefficient of variance expressed as a percentage on the Ct level, *coeff. of corr.* (*r*) correlation coefficients of each gene

## Conclusion

This is the first systematic analysis of the stability of commonly-used reference genes in human MSC. GeNorm, NormFinder and BestKeeper are freely available and easy-to-use Excel-based programs that are used to analyze the stability of reference genes. Of the eight reference genes, B2M and PPIA were identified as the most stably expressed genes in RT-qPCR analyses of human MSC. Despite its popular use as a reference gene for RT-qPCR analyses, GAPDH was ranked the least stable gene, and its extensive variability excludes it as a suitable reference gene in human MSC.

This study highlights the significance of reference gene identification by showing that reference genes can be cell-, tissue-, and experiment-specific. Here we offer a short list of reference genes as a starting point for researchers studying gene expression in human MSC and suggest that the widely available bioinformatics tools be used as an effective approach to identify and validate reference genes for each experimental condition.

## Electronic supplementary material

Below is the link to the electronic supplementary material.

Supplementary material 1 (DOCX 14 kb)
