# Supplementary material for: Identification of appropriate reference genes for human mesenchymal stem cell analysis by quantitative real-time PCR
Source: Biotechnol Lett. 2014 Sep 2;37(1):67–73. doi: 10.1007/s10529-014-1652-9 (PMC4279059; doi:10.1007/s10529-014-1652-9)
Supplement: Supplementary file 1 — Supplementary material 1 (DOCX 14 kb) [file 10529_2014_1652_MOESM1_ESM.docx]

**Supplementary Table 1. Summary of reference genes used in this study.**

| Symbol | Name | GenBank ID | Primer sequences | Product sizes (bp) |
| --- | --- | --- | --- | --- |
| 18S | 18S ribosomal RNA | NM10098.1 | F- GTGGAGCGATTTGTCTGGTT  R- AACGCCACTTGTCCCTCTAA | 115 |
| GAPDH | Glyceraldehyde-3-phosphate dehydrogenase | NM 002046 | F-ATGGGGAAGGTGAAGGTCG  R-GGGGTCATTGATGGCAACAATA | 108 |
| ACTB | Actin,beta | NM_001101 | F-GAAGATCAAGATCATTGCTCCT  R-TACTCCTGCTTGCTGATCCA | 111 |
| PPIA | Peptidyl-prolylisomerase A | NM_021130.3 | F-TCCTGGCATCTTGTCCAT  R-TGCTGGTCTTGCCATTCCT | 179 |
| B2M | Beta-2-microglobulin | NM_004048.2 | F-CTATCCAGCGTACTCCAAAG  R-GAAAGACCAGTCCTTGCTGA | 188 |
| RPL13A | Ribosomal protein L13a | NM_012423.2 | F-CGAGGTTGGCTGGAAGTACC  R-CTTCTCGGCCTGTTTCCGTAG | 121 |
| HPRT1 | Hypoxanthinephosphoribosyl transferase1 | NM_000194 | F-CCTGGCGTCGTGATTAGTGAT  R-AGACGTTCAGTCCTGTCCATAA | 131 |
| TBP | TATA box binding protein | NM_003194 | F-GCACAGGAGCCAAGAGTGA  R-GTTGGTGGGTGAGCACAAG | 174 |
